# Building back better from COVID-19: Knowledge, emergence and social contracts

**DOI:** 10.1177/03091325211059569

**Published:** 2022-02

**Authors:** Mark Pelling, Helen Adams, George Adamson, Alejandro Barcena, Sophie Blackburn, Maud Borie, Amy Donovan, Anshu Ogra, Faith Taylor, Lu Yi

**Affiliations:** 121212 King’s College London, London, UK; 6395 Oxford Brookes University, London, UK; 121212 King’s College London, London, UK; 2152 University of Cambridge, Cambridge, UK; 4616 King’s College London, London, UK; Sichuan University, Chengdu, China

**Keywords:** COVID-19, disaster, build back better, emergence, science and technology studies, social contracts

## Abstract

COVID-19 recovery is an opportunity to enhance life chances by Building Back Better, an objective promoted by the UN and deployed politically at national level. To help understand emergent and intentional opportunities to Build Back Better, we propose a research agenda drawing from geographical thinking on social contracts, assemblage theory and the politics of knowledge. This points research towards the ways in which everyday and professional knowledge cocreation constrains vision and action. Whose knowledge is legitimate, how legitimacy is ascribed and the place of science, the media and government in these processes become sites for progressive Building Back Better.

## I Introduction

A fair recovery for a world living with COVID-19 points to the importance of interactions between science, policy and public discourse and action. Informed by notions of emergence, codesign and of crisis as a constant, unfolding within development (Manyena, 2012), we offer a research agenda for recovery that foregrounds social processes of knowledge coproduction and identity formation as key mediators in shaping prospects for a socially progressive recovery (Jasanoff 2004). This approach applies analytical frameworks from the geographical literature on vulnerability, crisis and disaster recovery (e.g. Adger et al., 2012; Reyers et al., 2015; Anderson et al., 2019) to ask how the social consequences of COVID-19 and responses to it might help open opportunities for an inclusive Building Back Better. As a policy goal, Building Back Better moves success in recovery and reconstruction from returning to pre-disaster status to an enhanced status, both in terms of higher resilience and wellbeing. First introduced in 2006 by the United Nations Secretary-General’s Special Envoy for Tsunami Recovery, former US President Clinton, the ambition to Build Back Better has been incorporated into the UNDRR Sendai Framework for Disaster Risk Reduction 2015–2030 and into the United Nations Comprehensive Response to COVID-19 (UN 2020).

**Figure 1.**
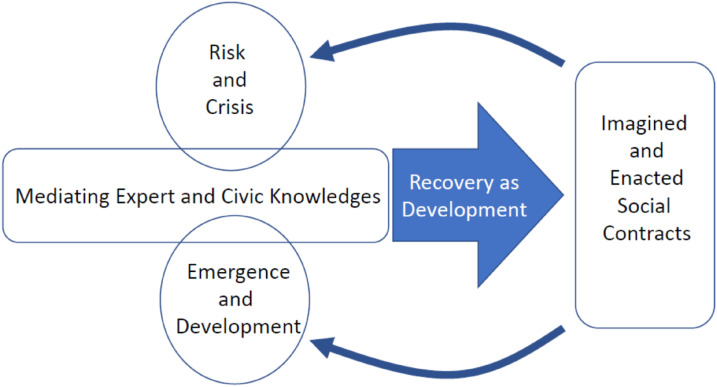
Recovery as development.

Recovery is understood to arise from actions and dynamic processes that have consequences for risk and development with knowledge construction and contestation running throughout (Figure 1). The COVID-19 pandemic as an open-ended crisis emphasizes the coevolving of cycles of risk, loss and recovery with development as knowledge, institutions, practice and the materialities of life (including the non-human). In this way, COVID-19 recovery is part of development, not a discrete period or agenda enacted externally to development. This perspective raises questions about the degree to which locally emergent response and recovery actions can persist or be scaled up through development, in planned and unplanned ways, and the consequences of this for the balance between rights and responsibilities in society. For example, will novel hand-washing stands opened by low-income and informal settlement dwellers contribute to wider development claims for improved access to basic services? Will voluntary neighbourhood associations filling gaps in individual and state capacity to care for the isolated at risk during COVID-19 progress into strengthened institutions for local solidarity or national reforms of social care?

Gramsci (1971) described political crisis as a moment of heightened tension between established and emergent forms of social organization. The COVID-19 crisis has helped to reveal the ‘fabric’ (Starn, 1971) of social and biosocial relations and offer a potential turning point in development (Koselleck, 2006). It becomes important then to observe the narrative forming practices and trajectories wrapped up in crisis (Roitman, 2014) as well as emergent social relations and political positioning (Anderson, 2021). Crisis events can destabilize the institutions and practices of governance (Collins, 2018), de-anchoring the integrity of power (McGowan and Donovan, 2021), opening scope for progressive, transformative change (Pelling, 2011), but more often to the reconfiguring, reasserting and extending of dominant market, governance and social relations (Klein, 2008). The agenda proposed here focuses on the relational arc from a state of pre-COVID-19 institutionalized stability, in which dominant social structures and social agency were reasonably well-aligned and reinforcing, through crisis-induced instability to emergence and the possibility of establishing a new (even if temporary) more progressive stability (Pelling and Manuel-Naverrete 2011) in human and more‐than‐human relations (Fraser et al., 2020). The analytical tool of the social contract is deployed to question the uneven working of power in the distribution of rights, responsibilities, protections and obligations in society (O’Brien et al., 2009) and how these might be reconfigured through disaster into response and recovery – asking recovery of what, for whom? Assemblage thinking is used to examine novel emergence across this tension, placing emphasis on the influence of diverse actors and actants (e.g. the virus, immunization, test and trace mechanisms) in the opening of sites through which the pre-disaster turns into the post-disaster (Donovan, 2017). Science and Technology Studies (STS) approaches directly address knowledge as contested and multiple, a product and determinant of individual and organisational authority that can directly shape recovery (Cooper and Praten, 2015), especially under conditions of uncertainty (Scoones and Stirling, 2020).

The emphasis on knowledge and identity alongside a critical materiality reflects the prominence given to science and its use or rejection by the more or less powerful in the COVID-19 pandemic. This helps frame inquiry into the (re)configuration of knowledge production (institutional forms, evidence bases for decision-making and popular discourse) as a product of and an active component in shaping the direction of recovery (McGowran and Donovan 2021). This approach is resonant with the UN Research Roadmap for the COVID-19 Recovery (UNSDG 2020) which calls for attention on both the technologies and the politics of knowledge coproduction for COVID-19 recovery. The UN Roadmap invites research to support Building Back Better, that will: ‘reimagine and rebuild health, social and economic systems so that they leave no one behind’ (ibid: 9). This vision maps onto the global recovery priorities identified by the UN Socio-Economic Recovery Framework for COVID-19 (UNSDG 2020) allowing governments and international agencies to repurpose the US$17.8 billion Sustainable Development Goal (SDG) programme and so operationalize the principle of leaving no one behind in the Build Back Better agenda for COVID-19.

To open up analysis of the COVID-19 pandemic beyond the statistical, we understand pandemic as socio-biological. An outcome not only of the spread, arrival and internalisation of previously external disease vectors but also of ongoing social behaviour and conditions that allow disease to take hold. This extends biomedical notions of pandemic that frame risk as external, to be responded to by top-down, fortifying barriers to transmission including national or local border controls, social distancing and robust surveillance (Dry and Leach 2010). The socio-biological opens options from social policy for a more integrative and comprehensive approach to pandemic avoidance and recovery – one that can directly inform long-term equity in development and point toward transformative recovery that can Build Back Better. Informed in particular by the experience of Ebola as a socio-biological emergency (MacGregor et al., 2020), recovery can consider social norms, behaviour and identity and underlying social and economic contexts, considering crisis and recovery as part of ongoing development, not exceptional to it (Anderson et al., 2019).

There is a long tradition of exchange between crisis, disasters and health policy and research. Perhaps, the most established connection points have been in the common metrics and methods for quantitative measures of the health impact of disaster (Tiernan et al. 2019). The inclusion of the biological and technological hazard alongside natural hazard risk in the UN Sendai Framework for Disaster Risk Reduction, 2015-2030 moved crisis and disaster policy towards recognising multiple dimensions of risk as systemic, demanding more focus on the interface between these fields of risk research and policy (UNDRR 2015). This has been able to draw on research on the social determinants of health vulnerability (e.g. Cutter et al., 2014) and the resilience of health systems (Francis and Bekera 2014) to disaster risk. Geographical research has been influential, including the use of a political ecology framework to help place health geography within unfolding processes of socio-ecological change (Jackson and Neely 2014). Still, aside from research on mental health (Goldmann and Galea, 2014), there has been little focus on event recovery, especially where health impacts cascade out into economic, social and environmental domains. Djalante et al. (2020) in reviewing the implications of COVID-19 for the Sendai Framework see opportunity in extending knowledge and science provision and sharing approaches from disaster risk governance particularly for humanitarian coordination and the strengthening of community level preparedness and response. Pandemic research, for example on Ebola, has considered social response and recovery but predominantly as a tool to reduce future outbreaks and build pandemic resilience (Green 2016), with limited comment on wider processes of development and recovery. In contrast, research on disaster risk reduction (Fernandez and Ahmed 2019), transformative adaptation (Pelling 2011) and sustainable recovery (Collodi et al., 2019) have increasingly focused on the messy transitions from recovery into development and from development into preparedness and on scope for leveraging progressive social change through these moments. The agenda presented here hopes to contribute to these conversations by bringing perspectives from crisis and disaster studies on recovery as a moment for progressive social change into COVID-19 recovery.

This approach raises questions about how recovery is understood, who the winners and losers of recovery might be, and how relationships between the public and state change before, during and beyond the pandemic. Three questions arise from this discussion which drive the following sections:

• How have particular *framings or deployments* of knowledge, including science and policy, impacted on specific segments of the population and their relationships to each other and the state, for what purpose, and with what exclusions?
• How has the *performance and presentation* of’ knowledge, including science, impacted on citizen expectations of fair governance?
• What lessons from the *use and communication* of knowledge, including science, can be learned to either strengthen or challenge public assumptions about acceptable governance, rights and access to social justice in recovery?

The following section describes the COVID-19 crisis and response to the point of paper submission. We then examine how recovery as emergence might be understood through the lenses of social contracts and assemblage, and with particular reference to the UK experience, consider knowledge, power and action in Building Back Better.

## II Disasters are multiple, crises are complex, context counts

COVID-19 was first recognised internationally on 31 December 2019, when the World Health Organisation was informed of cases of pneumonia of unknown cause in Wuhan, China. On 11 March 2020, WHO declared a global pandemic. Subsequent national response measures have included a combination of testing and treating patients, contact tracing, limiting travel, quarantining citizens, closing shops, restaurants and bars, and cancelling large gatherings (Bedford et al., 2020). Such measures curbed epidemic spread but also impacted on individuals, communities, and societies by bringing social and economic life to a near stop (Kupferschmidt and Cohen, 2020).

Inherited, pre-COVID-19 inequalities such as overcrowded conditions concentrated transmission risk in low-income urban centres (Khatua 2020) and in refugee camps (Banik et al., 2020). Added to this, lockdown policies have consistently worsened pre-existing social inequalities with a disproportionate impact on the mental health and physical wellbeing of children, care givers – many of whom are women – and some ethnic minorities (Proto and Quintana-Domeque 2021). Access to sexual and reproductive health services for women has been constrained (Dasgupta et al., 2020), and rates of intimate partner violence (which predominantly affects women) have increased due to stay-at-home policies and as the public sphere shrinks (Agüero, 2020; Mahase, 2020). Globally, women have carried a greater burden of loss of employment (Alon et al., 2020) and additional caring responsibiity (Zamarro et al., 2020; Alon et al., 2020; Micola et al., 2020; Bradbury-Jones and Isham, 2020). This is compounded by ethnic and racial differences in prevalence of COVID-19 which were quickly recognised to be related to structural inequalities in access to adequate housing, safe working conditions and economic security (Bentley, 2020). Going forward, COVID-19 impacts, responses and their consequences are likely to become a continuing aspect of life for some time, as locales and countries move in and out of containment and economic impact support measures with global economic effects. The World Bank estimated a 5.2% contraction in global GDP in 2020 alone. Economic volatility triggered by the pandemic are expected to lower investment, erode human capital through lost work and schooling, and fragment global trade and supply linkages (World Bank, 2020). Taken together, these well-documented determinants of unequal vulnerability and exposure demonstrate the need for transformations in social, economic and political life if recovery is to genuinely Build Back Better.

Information and its management have become central for governments seeking to manage COVID-19, while maintaining trust and consent, or at a minimum compliance, of the public. The rapid sharing of scientific information in-country and between countries has proven crucial for governments as a baseline to determine preferred science-based responses (Mitsuya and Kokudo, 2020). But the effectiveness and openness of data collection technology has been uneven across sectors and geographies and the degree to which science has informed policy has varied widely between countries – both due to differential scientific resources and political context. Excess mortality and unemployment have become the most prominent indicators of impact, both draw on readily available data, but simplify the crisis to a trade-off between health and the economy and away from the multiplicity of cascading consequences in education, mental health, culture, nutrition and the environment.

Information exchange for tracking and responding to COVID-19 has extended to smartphones, cloud computing and bigdata to set a new precedent for technological and data-driven approaches to understanding and managing risk in society (see Johns, 2020). These platforms allow individuals to make informed decisions and reveal information about susceptibility (such as particularly high prevalence of COVID-19 in poorer areas and areas with poor air quality, e.g. Fattorini and Regoli, 2020). However, these apps and the data (e.g. deaths, symptoms) conveyed also frame the COVID-19 pandemic as strictly medical. A more integrative socio-biological understanding of pandemic and recovery requiring a multi-sector response is not communicated via these platforms (O’Callaghan, 2020).

The technological and dashboard-driven approach to managing COVID-19 leaves little room for local knowledge, conversation, disagreement and a complex approach to the situation. At the local government level, impact maps and dashboards can become an evidence base that shuts down plural conversations, rather than opening up discussions about alternative futures for an area (Borie et al., 2019). Dashboard maps frame hotspot areas such as ‘infected slums’ as part of the problem rather than the solution. As O’Callaghan (2020) shows in a study of Ebola in West Africa, the success of response was made or broken by inclusion or exclusion of non-official actors (e.g. church groups, elders) and a bottom up approach. Online platforms can document the range of small, unofficial activities taking place – both to better understand, organise and improve access to these activities, but also to serve as a long-term testimony of the bottom up response to the COVID-19 pandemic and open up discussions about the future (Taylor et al., 2020).

These limitations indicate research on how technologies can better record and communicate the lived experience of pandemic, as well as the biomedical statistics describing health impacts. This might include research to make data structures and platforms flexible enough to document the broad impacts and response to Covid-19 beyond a strictly medical, quantitative framing. In the age of bigdata that is readily available to many it is also important to capture the contextual understanding of Covid-19 vulnerability, impacts and responses to help frame priorities for recovery.

## III Disasters, disruption and emergence

The mediating role of information technology and local knowledge in constructing popular, political and scientific imaginaries of crisis is constrained by surrounding institutional structures and social relationships (Fishbein and Ajzen 1975). In crisis, it is the interplay of knowledge creation with institutional or behavioural emergence that shapes the opportunity spaces for response and that begin to bound what is possible in recovery (Angell, 2014; Collodi et al., 2019). Understanding the relational processes through which these spaces are given meaning and by who, is the first step in tracking socially progressive or regressive trends that might shape recovery. Here we review two entry points for understanding the opening of political space during crisis and into recovery. Social contracts draw from classical political theory and more recent interpretations of power struggles between social structures and actors in political economy and political ecology (Blackburn and Pelling 2018). Assemblage Theory brings post-structuralist thinking to emphasize the ways in which power is materialized through relationships that need not be hierarchical nor entirely human (Deleuze and Guattari 1988; DeLanda, 2002, 2016). There are epistemic differences between these schools built on assumptions of scalar and flat ontologies. However, both approaches reveal the importance of social trust in maintaining stability and how spaces for novel political action can arise through the breaking and potential reformulating of social relations through crisis. Together, these entry points help interpret the ways in which knowledge flows through and shapes relationships and resulting spaces for change.

### 1 Disrupting trust and social contracts

Social contracts are upheld by the legitimacy of knowledge production processes that can shape development and crisis management policy. They depend on the authority of institutional bodies to represent the collective and define the rights of a socially differentiated public. Citizens (hypothetically) collectively consent to the powers governing them, in exchange for the protection of their rights (Campbell 2010). Social contracts describe what distribution of rights-responsibilities is considered acceptable and fair, and hence define development visions. They may be enshrined in law (e.g. we pay tax to fund public services), but not necessarily.

Crises and disasters are known to make visible and magnify gaps and inequities in state service provision and citizen protection – surfacing questions around the adequacy and legitimacy of risk governance (Pelling 2011). Whilst social contracts often demonstrate resilience in crisis (Siddiqi and Canuday 2018), where disaster losses are deemed socially unacceptable and political legitimacy is lost, an opportunity space can arise for governance norms to be questioned – and if captured politically, for a new or evolved social contract to emerge (Christoplos et al., 2017; Pelling and Dill 2010). It has been suggested that government failings during COVID-19 have revealed deficiencies in the assumed social contract between state and citizen, for example, in failing to secure personal protective equipment for health and social care staff and building adequate testing facilities (The Lancet, 2020).

Here, we argue that social contracts open questions around post-COVID renewal in two ways. First, the pandemic demonstrates how different experiences of COVID-19 are shaped by existing differences in the relations between state and society (e.g. in groups’ ability to enact full citizenship due to race, class, citizenship status) and each other (e.g. through racial, gender and border dynamics that structure employment opportunities) – indicating multiple, differentiated social contracts through which diverse marginalized groups seek out and secure rights protections, beyond the formal state. Indeed, the classical contractarian principle of a single, unified social contract based on freely bargained rights or sovereignty has been rejected (Boucher and Kelly 1994). Such a view has allowed dominant social contracts to protect structures of exclusion and oppression, by assuming free bargaining of rights or sovereignty by and for the European white male (Pateman and Mills 2007).

Second, a social contracts lens invites interrogation of the conditions under which state legitimacy is shaken and the limits to public trust in authority (Adger et al., 2012). COVID-19 highlights in particular the role of knowledge as a mediator in social contracts (for example, publics consent to lockdown or track and trace surveillance because they trust the policies are based on is confidential, accurate data that is utilised in good faith). As addressed in Section IV, access to scientific knowledge is uneven and political. Science is one form of knowledge alongside multiple diverse public knowledges that are non-technical and produced through grounded, everyday experience, for example, about symptom variability, infection rate, the enforcement of track and trace. Where trust in authority’s use of science wavers, for example, after the government ‘u-turn’ on the use of face-masks in the UK, or the apparent disregard of official advice by officials themselves (e.g. the chief adviser to British Prime Minister Boris Johnson, Dominic Cummings’ controversial visit to Barnard Castle during UK lockdown in April 2020), alternative social contracts may come to the fore based on those alternative knowledges. The politicization of scientific knowledge has come under close scrutiny under COVID-19, and the pandemic has seen people stepping outside their normal relationship with authority and with science, both through non-compliance with regulations and in the myriad spontaneous, local responses to COVID-19 in the community. Clashes between differentiated social contracts, mediated by differentiated knowledges, may exacerbate or widen existing social-political gaps.

In highlighting these diverse and dynamic social contracts, mediated by diverse and dynamic risk knowledges, COVID-19 highlights the need for a critical social contracts analysis focused on unpacking what kind of protections are afforded by what kind of social contract, to whom and where (O’Brien et al., 2009). Such a lens demands attention to the power dynamics that underpin disjunctures between imagined, legislated and practiced social contracts, and the pathways through which certain visions of fair/just governance become dominant (i.e. fixed in mainstream political discourse, law and practice) over others (Blackburn and Pelling 2018). This analysis must go beyond state-society relations and also consider social contracts with/among non-state actors engaged in service delivery (e.g. private sector, NGOs, faith or community groups) (Zadek 2006; White 2007), as well as social contracts between society and science (Gibbons 1999) and the more-than-human (Serres 1995; Whatmore 2002). Understanding multiple social contracts demands attention to diverse knowledges produced through intersecting social relations.

Key questions include: to what extent has COVID-19 ruptured dominant social contracts? What alternative social contracts have emerged or reconfigured during and after COVID-19, how and why? And what is the role of scientific and other knowledges, including scientific uncertainty, in this reconfiguring? Assemblage provides a useful lens on the fluid construction and contingent quality of these relationships – as outlined in *Breaking and reformulating assemblages through disaster events*.

### 2 Breaking and reformulating assemblages through disaster events

The materialisation of the COVID-19 pandemic, with its resulting human and economic losses, psychological stresses and disruptions of social relations was already present as an incipient possibility before it happened. In considering post-disaster recovery as an opportunity to reduce future risk, critical disaster studies have argued for an historical analysis of underlying causes of vulnerability, often emphasising the role of hierarchically scaled institutions (Blackburn, 2014; Marks and Lebel, 2016; Wisner et al., 2004). Extending from this literature, work drawing on Assemblage theory (DeLanda, 2002, 2016; Deleuze, 1994; Spinoza, 2000) argues that a historical lens can also reveal the unexpected potential for change that arises from relational ruptures generated by a crisis (Barcena, 2021; Donovan, 2020; Marks, 2019; Ranganathan, 2015). This work sheds the assumption of vertically organised institutions, and rather conceptualises risk production as a horizontally networked, fluid mesh of social, economic, ecological and political relations.

In relation to COVID-19 renewal, this approach invites the question of how ruptures in social relations have transformed the space of political possibilities, and how such emergent potentialities might reduce, increase or redistribute risk of future pandemics. Assemblage thinking offers responds to this question by focusing on the interplay of self-organising processes that emerge in response to the material and affective disruptions ensuing a pandemic. This is not to disregard how hierarchical relations of power flow vertically along institutional landscapes, reproducing conditions of vulnerability, but to open the analytical gaze to emergent processes of organisation ablility to disrupt and transform (enhance, shift as well as reduce) the production of vulnerability.

The concept of geo-event (Shaw, 2012) is helpful to conceptualise how social contracts, knowledge production processes and risk-development visions are opened to unexpected change triggered by disruption from a pandemic, or a disaster in general. A disaster disrupts the space of possibilities of individuals in which they rehearse the relationships from which they draw meaning and from which they make sense of their context, their history and themselves (Harman, 2002). In this way, Shaw (2012), drawing on Deleuze, suggests that affective relationships precede cognitive understandings. The disruptions triggered by a disaster, therefore, can break existing assumptions and open the possibility of transformed ideas and relationships, and with them, new understandings of the world and of oneself.

During the first few weeks of the pandemic, a burgeoning number of mutual aid networks emerged globally with the objective of responding to economic and social needs of urban residents, who at same time saw their bonds with employers, families and relatively distant friends restrained (Kavada, 2020; Pleyers, 2020). Examples of local social action involved very different activities, including the provision of food and medical goods, the production of masks; the offering of support services such as schooling, so economically deprived parents could continue working; psychological support for those affected by anxiety and domestic violence; and emergency transport to hospitals (New Socialist, 2020; Pais, 2021; Travlou, 2020; Woods, 2020). This emergence illustrates how the geo-event of COVID-19 triggered transformations in the space of possibilities where relationships were being materialised. However, work has yet to ask what the preconditions were for such emergence and how these might be fostered, or how far such progressive acts might be a vanguard for a wider progressive recovery.

Locally progressive acts do not happen in isolation from wider political considerations. Members of these networks have to determine whether they should supplement the strained capacity of the Government to fulfil its part of the social contract during the extreme circumstances of the pandemic or demand the fulfilment of the Government responsibility (Jun and Lance, 2020; O’Dwyer et al., 2020). This is particularly strained where the political branding and sponsoring of these groups has caused internal tensions (New Socialist, 2020). These debates rehearse pre-pandemic political positions. However, Shaw (2012) has shown that it is in the relationships established in response to a geo-event that one can find the seeds for the dislocation of dominant understandings of the world.

Understanding the emergence through crisis of particular ways of thinking and acting, demands a focus on the processes by which relationships are freed, established anew and potentially recaptured by political interests. Woodward and colleagues’ (2012) idea of ‘site’ helps to illustrate this concern. The concept of site sheds light on the constant negotiation undertaken by members of a network, or any other social arrangement, to maintain and repair its constitutive relations and ordering structure (Spinoza, 2000). But also, it implies examining the processes that erode these relationships (Schatzki, 2002; DeLanda, 2002; Bonta and Protevi, 2004). As a result, the future is opened for the establishment of new relationships, which indeed harbour the possibility of imagining, asserting and potentially institutionalising new social contracts. In this way, risk-development trajectories result from the assembling processes that incrementally stabilise and destabilise social orders, capturing and freeing individuals. This places a crisis like COVID-19 as a destabilising event with the potential to alter the risk-development trajectory of a place.

In this line, COVID-19 has provided some of the conditions of possibility to advance existing alternatives to dominant social contracts. For instance, feminist groups have brought to the attention of the public the asymmetric effects of COVID-19 (Roesch et al., 2020). These groups have highlighted increased incidence of domestic violence on women by men during lockdown, the asymmetric exposure of women to COVID-19 because of their more frequent presence in supermarkets, and the asymmetric economic impact on domestic workers, frequently women, under informal work arrangements (Yearby and Mohapatra, 2020). More widely, health workers have mobilised globally to denounce the damaging effect of austerity politics over health systems, which in turn has increased social vulnerability to COVID-19 pandemic (Pleyers, 2020).

An analytical approach that considers emergence should examine the coevolutionary relationship between self-organising processes and extant relationships (Barcena, 2021). Responding to the question of how networks develop the capacity to transform demands an understanding of preceding disassembling processes. This invites an analysis of how individuals identify, consolidating social networks and how networks interact and contribute to changed collective behaviour and social norms. Attending to the creation of new relationships, this framework can also approach the question of how alternative expectations and institutional roles are stabilised, and with them, alternative configurations of winners and losers are established during recovery. For instance, the kinds of institutions that can provide spaces to consider how conflicting ideas of justice can be resolved based on emergent and competing moral frameworks.

## IV Knowledge, power and action to build back better

The preceding discussion has identified ruptures in social contracts and the qualities of emergent assemblages as entry points to Building Back Better. Here, we focus on knowledge as mediating rupture and emergence and as a point of rupture in its own right. Shocks such as COVID-19 refocus national strategies for the use of knowledge in policy-making by producing civic epistemological ruptures – they challenge and change how knowledge is tested and used in decision-making (Donovan and Oppenheimer 2016). In this way, ruptures in knowledge systems are tightly coupled with, can catalyse or be a consequence of, rupture and emergence in wider social relationships. We focus in this section primarily on the relationships between government, society and science, recognizing that each of these is a complex and plural entity.

COVID-19 has highlighted the importance of influential, competing knowledges in shaping action from individuals, businesses and governments. Information presented as factual by some has been undermined by others, generally building on different evidence and modes of interpretation – but also in line with ideological priorities. Multiple forms of knowledge and expertise have been mobilised in COVID-19 narratives, associated with different responses – a not uncommon observation in the interplay between knowledge, politics and action on risk and development (Leach et al., 2010). Incomplete knowledge has been woven into political ideologies and value systems; and the global media has provided at least as many accounts as there have been articles and podcasts. Analysis of these divergent narratives suggests that the crisis has been assembled out of a complex array of human and non-human agents (including the virus itself), perceptions, ideologies and relationships.

Building on STS literature on expert advice, we view science and policy as co-produced (Jasanoff 2005): as expressive elements of an assemblage that emerge from its constituent parts and interact with them, reconfiguring the assemblage as knowledges evolve. We argue that both science and the policies that emerge, haphazardly, from it are complex and value-laden with a role for science in policy, in particular through the provision of what has been termed regulatory science (Irwin et al., 1997) or serviceable truth (Guston 2001). Under this perspective, science is performative (Hilgartner, 2000), that is, it has influence on the ways in which particular issues are addressed, and experts need to be held accountable. One of the main concerns is that experts make decisions rather than feeding into a democratic process (Stirling 2010). In other words, scientific advice must be accountable to democracy (Beck, 1992; Brown, 2009). This also requires communication with publics – science is not fact, science is multiple: it is produced both by systematic scientific method and by social and political contexts, and it is interpreted in those contexts (Gieryn, 1983; Woolgar and Latour, 1986; Jasanoff, 2005). Conversely, however, politics should also be accountable to the scientific evidence – it should not be allowed to pick and choose its evidence to fit its own ends via the political strategic model (Owens, 2015). The multiplicity of science emerges not only from the multiplicity of scientists, but also from the wider political and economic contexts that fund and legitimize it.

### 1 Science on the inside

Although scientific advice is routinely used in policy, in more or less formal ways (e.g. calls to develop evidence-based policy-making), COVID-19 is an example of expert advice in times of crisis (Donovan, 2021). In this context, expectations of what science should or should not produce and how governance should or should not work with science are made visible. To some extent, the COVID-19 crisis is also a crisis of expert advice, for example, outside institutionalised and long-established expert bodies, several ad hoc expert committees (e.g in France and the UK) and self-appointed knowledge brokers have been established to deal with the claimed exceptionality and urgency of the situation. The response-into-recovery period is a critical juncture for the relationship between science, governance and wider society, and the influence of institutional change in this period is likely to be long-lasting.

While some may argue that, in times of crisis, politics are suspended, analysis of the discourses of powerful political elites suggests instead that COVID-19 provides an opportunity to conduct politics by other means. Countless examples exist: Boris Johnson arguing that he will keep shaking hands (Reuters 2020a), might be interpreted as having performed a ‘strong and stable’ UK – and the UK’s reluctance to participate in EU procurement could be interpreted as Brexit politics. In Turkey, nationalist President Erdogan publicly dismissed a proposal by Istanbul’s mayor to enforce confinement – an act which performed the ongoing clash of ideologies between Erdogan and Ekrem Imamoglu (Reuters 2020b). US President Donald Trump’s criticism of the World Health Organisation, and decision to withdraw US funding, on claims of its weak response to COVID-19 is another example (New York Times 2020). If these events act as useful reminders of the persistence of politics and geopolitics, highlighting where States see themselves on the worldmap, there are at the same time representatives of different political projects and socio-technical imaginaries, defined as “collectively imagined forms of social life and social order reflected in the design and fulfilment of nation-specific scientific and/or technological projects” (Jasanoff and Kim 2009:119). Resonating with Anderson’s ‘Imagined Communities’ (1983), this concept emphasises how science and technology are themselves constitutive of particular dominant and alternative/emergent political projects, and visions of the future (e.g. Jasanoff and Kim 2009). The diverse ways in which nation-states have responded to the pandemic and the balance of priority placed on health/economic/social impacts is a case in point (Jasanoff et al., 2021). It is not only states that make knowledge claims and bend science to their interests (or are influenced by it). Jasanoff (2005: xy) responds to this through the notion of civic epistemology: ‘the stylized, culturally specific ways in which publics expect the state’s expertise, knowledge, and reasoning to be produced, tested, and put to use in decision-making’. This too helps place emergent knowledge and meaning more broadly within circuits of governance than government.

As with Shaw’s (2012) sites of emergence, these concepts highlight the importance of historical and cultural trajectories to account for the diverse ways in which what counts as authoritative expertise is institutionalised and received by different publics. In explaining spatial variations, they illustrate (i) the importance of context to identify how expert advice can help Build Back Better (albeit focused on national levels) and (ii) the challenges associated with changing long-established ways of dealing with knowledge (e.g. Forsyth 2019). Although disasters can be approached as moments of ontological disturbance (see Tironi 2014) where existing social contracts, solidified in assemblages and civic epistemologies, may be re-negotiated, achieving long-lasting changes in those arrangements remains incredibly challenging. While we argue that COVID-19 opens up different windows of opportunity, including changes in the use and provision of expert advice to Build Back Better, there are also numerous instances of responses to crisis mobilising science and expertise that reinforce pre-crisis power arrangements, leaving out alternative pathways and possibilities for change (Rhiney 2018). What has been called path-dependency in the literature on policy change is also largely explained by the existence of long-established civic epistemologies (Miller 2008; Adamson et al., 2018).

Ontologically, these questions are about the social relations – or social contracts – between science advisors, policymakers, politicians and laypeople, and about the role of other kinds of agents such as the virus itself, the virtual (internet claims and imaginaries) and the institutional (Donovan 2017). The view that any single person has of these relationships will differ and will be dynamic. People have positionality – particularly those in positions of power. During COVID-19 in the UK’s Science Advisory Group for Emergencies (SAGE), these relationships have been hidden, the covert nature of the scientific advice precluding both public trust and accountability. As the UK government faultingly released information in response to calls for transparency, public attitudes to and demands for expert advice have shifted: the civic epistemology of the UK is in a state of flux, or rupture, ripe with emergent potential.

### 2 The politics of expertise

How can scientific advice be constituted differently in light of COVID-19? Previous research on the topic of expert advice in disasters has raised four questions (Donovan and Oppenheimer, 2016; Donovan 2021): Locating expertise, representing expertise, contextualising expertise and governing expertise. All of these questions influence the trust and legitimacy of expert advice in a crisis. Incorporating and thinking through these dimensions will be important in practically Building Back Better how we use knowledge in policy.

How advisors are selected is important – both in terms of the ways in which scientists are chosen and the different disciplines and sectors that they represent. The UK’s SAGE has tended to incorporate very limited disciplines, focusing almost exclusively on epidemiology and behavioural science. These disciplines can give the illusion of simple answers and ignore many of the nuances of the pandemic-in-society that will be familiar to many social science disciplines. The focus on individual decision-making inherent within behavioural science also reflects a libertarian political ideology that is attractive to many governments. SAGE has tended not to take advice from international institutions like the WHO (which does incorporate a wider range of expertise) or to work with the EU, instead taking a Westphalian approach to disaster management. International bodies such as the WHO are not a panacea in terms of expertise, but they do use a wider range of disciplines in their work, and they include experts from many different countries. While there is clearly a level of institutionalisation involved in the resulting advice, it is less attached to the politics of individual nation-states.

Mechanisms for science reporting include questions of the representation and communication of uncertainty and how to communicate to diverse audiences where necessary. Transparency is important; repeated studies have demonstrated the value of open science in both increasing popular scientific literacy and in building public trust. This relates to the representation of diverse scientific tools. In the UK, there has been a heavy dependence on scientific models – one in particular – but a model is a simplified representation of reality built on a multitude of assumptions. The influential model developed by Imperial College, for example (Ferguson et al., 2006, 2020), was originally developed for pandemic influenza and incorporated data from the decade old 2010 census. It also had to make numerous assumptions about the nature of COVID-19. Yet, most of this information was only available for those who read the scientific literature – it was not in the popular, public domain. More recent SAGE reports have tended to use three sets of models, with very different results and input parameters (and little justification for the latter, at least in the documents released by government). Modelling is not, however, the only way to organize knowledge about pandemics – there are important insights both from the qualitative social sciences and from virology, for example, that are oversimplified in models. Communication of model results and the simplification of concepts such as the R number has provoked a particular scientization of the pandemic that shuts down the discussion of more socially nuanced topics (such as support for isolation). The ways in which science has been represented in this pandemic in the UK, then, are very narrow. This has an impact on wider social perceptions.

Popular as well as professional audiences will have different expectations of science and this influences how scientific and expert knowledge is received, and how far it might be acted upon. Many people hold an idealised view of science (Cassidy 2020). Where expertise appears to suggest a course of action guided by a particular political ideology, criticism therefore arises not from a general distrust of science, but the idea that these particular scientists must be corrupted (Cloud 2020) by money, influence or their own political agenda (Eiser et al., 2009). This ignores the reality of much scientific advice – particularly in areas of deep uncertainty and high social implications, where a) scientists' interpretations of uncertain and sometimes contradictory models and observations, and b) policy-makers’ decisions on how to act on scientific advice, are inherently political and value-laden (Owens, 2015; Fischer, 2011). In a situation where publics hold a widespread misapprehension about the nature of scientific advice, accountability can lead to populism and partisan attacks on experts (as has been witnessed in the UK in the pandemic). Accountability must therefore be accompanied with a drive to increase understanding of the realities of science and scientific advice. The simplest intervention here would be in compulsory school science syllabi.

The contextualisation of scientific advice is also important – and this includes how other kinds of knowledge are represented and positioned alongside scientific evidence. This is particularly important in plural societies – and something that the concepts of civic epistemologies and socio-technical imaginaries do not adequately capture if they focus solely on nation-states as single entities. Everyone involved in the pandemic – from government to essential workers – has values that influence how they appraise advice and make decisions in their lives. Scientific advice has not only to be relevant, but to incorporate an understanding of the complexity of social values and contexts in a plural society – as has been noted in the literature around public understanding of climate change, for example (Hulme, 2008, 2009). Without this science can simply be used to empower dominant actors justifying policy that excludes the marginalised, or be received as such, soliciting resistance and closing down opportunities for the emergence of transversal social collaboration to Build Back Better. Science is itself vulnerable – both to the limitations of funding agency expectations and scientists themselves, and to interference from politics. It contributes to the vulnerability of citizens, too, because of its own vulnerability to abuse and misuse – including the over-representation of some elements at the expense of others, and the use of uncertainty to obscure particular ideological choices.

The complex dynamics of expert advice, monitoring technologies and uncertain, incomplete datasets have been realised through COVID-19 as prevailing expectations and relationships between ‘science’ and the public come under strain. Science can have immense power in driving policy but also as a rhetorical device to be called upon and even scapegoated by politicians. Scientific resources are unequally distributed geographically, as are mechanisms for observation, experiment and interpretation. Fundamentally, however, the deployment (or not) of scientific advice in a disaster like COVID-19 can shift the relationships between science and the public in a civic epistemological rupture, as trust breaks down and the mechanisms for integration of knowledge and policy are shaken. This presents an opportunity for research and action in reorienting these relationships. Within the UK, this might lead to the inclusion of a wider range of disciplines on formal advisory groups like SAGE (there is an urgent need for involvement of social sciences like human geography, which can also provide some level of epistemological diversity on an advisory panel, and better represent a range of social values); other opportunities might be the inclusion of lay members on central or local government advisory groups – both for transparency and democratisation of advice - and especially as response leads into recovery. Within recovery, there is growing evidence from humanitarian action of the advantage of including survivors in visioning and planning (Collodi et al., 2019; Murphy et al., 2018). Increased interest in the science-policy-society relationship also presents opportunities for a wider discussion on the nature of science, science advice, and the relative strengths and weaknesses of different epistemologies. Ultimately, there needs to be a greater emphasis in Building Back Better on how scientific and other knowledges are deployed, institutionalised and interact with dominant and alternative policy narratives in crisis contexts.

## V Conclusions

Recovery from COVID-19 is unfolding amidst ongoing crisis as we adjust to a world-with-COVID-19. This complexity is testing and destabilising extant social norms and institutions, presenting opportunities for progressive transformation from pre-COVID-19 pandemic social relations and expectations. This moment is characterized by changes that are strategic and planned, and those that are emergent and unplanned. Understanding in this context how specific opportunities for change arise, which are realized and who benefits from success or failure is a core concern for critical studies and policy action of our time. To help open this research agenda we turn to and apply analytical frameworks already shown to be useful in understanding the arc of instability, emergence, competition and re-stability within studies of disasters and crisis including social-ecological systems transformations associated with climate change.

Drawing together work on social contracts, emergence and knowledge coproduction helps to extend research from accounting for the outcomes of change (how effective a specific policy or social action is), to uncovering processes of change making (how planned and emergent modalities interact and how far they might lead to more inclusive and sustainable processes of transformation). Placing specific acts or projects within the wider framing proposed here – of social contracts, emergent social (and more‐than‐human) relations and processes of knowledge coproduction – also helps to extend research on transformation into the moveable bio‐social contexts within which potentially and actually transformational actions arise and are shaped or curtailed. This helps to move past one impasse for transformative actions which will always be dependent on the viewpoint of the observer.

By better understanding the ways in which social relations are transformed (or not) in the ongoing recovery-crisis that is the COVOID-19 pandemic, the transformation opportunity space itself can be mapped, revealing sites of struggle over power that lie in local neighbourhoods, across organizational networks and in the shifting alliances and tensions between competing and collaborating interests. The flat ontology of this approach allows a break from accounting for change as reliant on only a top-down process. The proposed research agenda can help to identify the balance of innovation and control across multiple local sites and their connections to the state. For example, online platforms could be used to document the range of small, unofficial activities taking place already – both to better understand, organise and improve access to these activities, but also to serve as a long-term testimony of the bottom up response to COVID-19 and open up discussions about the future.

Knowledge is central to this perspective. Knowledge lends relationships meaning and is a site of contestation and potential emergence. As knowledge is cocreated expert and civic knowledges can be in tension, demonstrating sharply the politicized nature of science in the public arena. Part of Building Back Better in this context includes working on the institutions that connect science and policy so that political decisions can be more transparent in explaining why they deviate from science advice. Understanding the role of multiple sciences at the same time would require us to rethink how we comprehend science as a body of knowledge. Seen as a continuum, the nature of scientific advice can neither be purely deterministic, as expected of vaccine developers, nor can it be purely probabilistic, like models based on assumptions (which can potentially be manipulated) guiding policy action on the ground. Is there scope in this context to reimagine the role of the media in the world-with-COVID-19? With the advent of new media technologies in the last 20 years, fake news has proliferated. Might Building Back Better from COVID-19 open an opportunity to reconfigure media responsibilities in science and polity? Would this require legal frameworks? Can Building Back Better from COVID-19 include support for institutions that can make transparent and generate public accountability for how knowledge is shaped and shapes sites of emergence as well as how we interact with, co-produce and evaluate knowledge in society?
